# Evaluating Intravaginal and Transvaginal Electrical Stimulation Techniques for Urinary Incontinence: A Systematic Review with Meta-analysis

**DOI:** 10.5152/eurasianjmed.2026.251298

**Published:** 2026-10-16

**Authors:** Júlia Vitoria Souza Nazareth, Julia Ciriaco da Silva, Erick Andrade Lopes, Alberito Rodrigo de Carvalho, Márcia Rosângela Buzanello, Gladson Ricardo Flor Bertolini

**Affiliations:** 1 Western Paraná State University (UNIOESTE), Paraná, Brazil.

**Keywords:** Electric stimulation therapy, pelvic floor, urinary incontinence

## Abstract

**Background:**

This study aimed to determine the effectiveness of intravaginal electrical stimulation for managing urinary incontinence.

**Methods:**

Searches were conducted across PubMed, Embase, Cochrane Library, Scopus, Web of Science, Latin American and Caribbean Health Sciences Literature, and gray literature sources including Google Scholar, OpenGrey, and ProQuest. Risk of bias was evaluated using the Cochrane tool. The primary outcome was pelvic floor muscle (PFM) pressure, while secondary outcomes included PFM strength, involuntary urine loss, frequency of urinary leakage episodes, and quality of life.

**Results:**

Seven randomized controlled trials met the inclusion criteria, comprising a total of 505 women. Two of the included reports (Amaro 2005 and Amaro 2006) derived from the same patient population were treated as a single study for the purpose of this review. The pooled analysis showed significant improvements in PFM pressure (3.92 cm H_2_O, 95% CI, 1.50-6.34; *P* = .001; *I*^2^ = 55%), PFM strength (0.89, 95% CI, 0.37-1.41; *P* = .0008; *I*^2^ = 52%), frequency of urinary leakage episodes (−0.41 episodes/day, 95% CI, −0.78 to −0.03; *P* = .03; *I*^2^ = 73%), based on conversion of diary data to a common daily unit. No significant effects were observed for involuntary urine loss (3.68 g, 95% CI, –13.01 to 20.38; *P* = .67; *I*^2^ = 97%) or quality of life (standardized mean difference = 0.28, 95% CI, –0.42 to 0.98; *P* = .43; *I*^2^ = 85%).

**Conclusion:**

Intravaginal/transvaginal electrical stimulation appears to enhance PFM pressure and strength and reduces leakage episodes in the short term. However, its effects on objective urinary loss and quality of life remain inconclusive, and the evidence is of very low certainty.

**Main Points**

- This systematic review and meta-analysis included 7 randomized controlled trials comprising 505 women, evaluating intravaginal and transvaginal electrical stimulation for urinary incontinence.
- Electrical stimulation significantly improved pelvic floor muscle pressure (3.92 cm H_2_O; *P* = .001) and strength (0.89; *P* = .0008), with low-certainty evidence.
- A modest reduction in the daily frequency of urinary leakage episodes was observed (−0.41 episodes/day; *P* = .03) though the evidence was rated as very low certainty.
- No significant effects were found for objective involuntary urine loss (*P* = .67) or quality of life (*P* = .43), both with very low certainty evidence.
- Although electrical stimulation shows short-term promise for surrogate measures of pelvic floor function, its clinical benefits on incontinence severity and quality of life remain uncertain, and substantial heterogeneity precludes definitive clinical recommendations.

## Introduction

Urinary incontinence refers to the unintentional release of urine and represents a widespread health issue, particularly among women. Proper function of the pelvic floor muscles (PFMs) is crucial for continence, as these structures provide support to the bladder and urethra. When pelvic floor integrity is compromised—whether through aging, increased physical load, or vaginal childbirth—urinary leakage may develop. Several forms of incontinence exist, including stress urinary incontinence (SUI), typically triggered by activities that raise abdominal pressure, and urge urinary incontinence (UUI), which involves a sudden, compelling need to void.^1^^,^^2^ Urinary incontinence is a condition that, although it does not directly affect mortality, causes significant psychosocial changes in women.^3^^,^^4^ The prevalence is quite variable, between 25% and 45%, with around 40% of women over 70 suffering from this condition.^5^

Treatment for urinary incontinence is wide-ranging, from lifestyle changes and behavioral changes to more aggressive interventions such as medication, neuromodulation, pelvic floor strengthening exercises, the use of mechanical devices, and surgical treatment.^6^^,^^7^ Other conservative physiotherapeutic approaches include intravaginal electrical stimulation (IES) and transvaginal electrical stimulation (TES). Both modalities are designed to enhance PFM function and alleviate symptoms of urinary incontinence, providing practical, low-risk treatment options with minimal adverse effects.^8^^,^^9^ Intravaginal electrical stimulation can be effective in both SUI and detrusor instability, helps to reduce involuntary contractions, and improves patients’ quality of life.^10^ According to Barroso et al,^8^ this also applies to TES, which is a viable alternative for treating the main forms of female urinary incontinence, providing symptom relief and improving patients’ quality of life. Given these positive reports, this study aims to search the literature for the state of the art on the effectiveness of these therapies in patients with urinary incontinence (UI).

## Material and Methods

### Design

This systematic review was carried out and reported following the guidelines of the Preferred Reporting Items for Systematic Reviews and Meta-Analyses (PRISMA). The protocol was prospectively registered in the Open Science Framework under the DOI 10.17605/OSF.IO/8YF4S , on March 18, 2025. Since this is a literature review, approval by an ethics committee and informed consent is not required.

### Eligibility Criteria

The population intervention comparison outcomes study design framework was used to define the research question for this study: P (population): women with UI; I (intervention): intravaginal or TES; C (comparison): placebo or no intervention; O (outcomes): primary outcome—PFM pressure; secondary outcomes—PFM strength, involuntary urine loss, frequency of urinary leakage episodes, and quality of life; S (study design): randomized clinical trials.

Inclusion criteria: women with UI, aged over 18, complaining of urinary loss during physical activity, activities of daily living, or episodes of urinary urgency. Studies without time or language limits were included.

Exclusion criteria: women with other pelvic dysfunctions and pregnant women. Studies for which the full text was unavailable were also excluded.

### Sources of Information

The indexed databases searched included PubMed, Web of Science, Embase, Cochrane Library, Scopus, and the Latin American and Caribbean Health Sciences Literature. Gray literature was explored through Google Scholar, ProQuest, and Open Grey.

### Search Strategy

Keywords were selected from the Medical Subject Headings and Health Sciences Descriptors via the Virtual Health Library. The search terms used were: “Urinary Incontinence” AND “Electric Stimulation Therapy.”

### Selection of Studies

References were managed and duplicates removed using Rayyan Qatar Computing Research Institute and EndNote Web software.

The records were screened by 2 independent reviewers (R1 and R2) in 2 stages. In the first stage, titles and abstracts were assessed based on the eligibility criteria. In the second stage, the full texts were reviewed, and articles were selected according to the same criteria. All extracted information was subsequently verified. In cases of disagreement at either stage, a third reviewer (R3) was consulted to make the final decision.

### Individual Assessment of the Risk of Bias in Studies

The risk of bias was evaluated using the Cochrane Risk of Bias tool (ROB 2) by 2 blinded reviewers (R1 and R2), with any disagreements resolved by a third reviewer (R3). Each included study was assessed across 5 domains: deviations from intended interventions, missing outcome data, outcome measurement, selection of reported results, and overall bias. Each domain was classified as having a low risk, some concerns, or a high risk of bias.

### Methanalysis

Statistical analyses were performed using RevMan 5.4.1 (The Cochrane Collaboration, Oxford, UK).^11^ Continuous outcomes were expressed as standardized mean differences (SMDs) with 95% CIs, and a *P* < .05 was considered statistically significant. Heterogeneity among studies was assessed using the *Q*^2^ and *I*^2^ statistics. *I*^2^ values of 0%-40% were considered potentially unimportant, 30%-60% indicated moderate heterogeneity, 50%-90% substantial heterogeneity, and 75%-100% considerable heterogeneity.^11^ A random-effects model was applied when significant heterogeneity was present. When required, data transformations were performed according to the Cochrane Handbook, Chapter 6.^12^

Because Sand et al^13^ reported PFM pressure in mm Hg while the other studies used cm H_2_O, values were converted to a common unit (1 mm Hg = 1.36 cm H_2_O) before pooling. This conversion was applied prior to the meta-analysis; standard errors were adjusted accordingly. To explore the potential impact of control group type, subgroup analyses were planned a priori for the primary outcome (PFM pressure) and conducted post hoc for secondary outcomes, grouping studies into those using sham/placebo controls vs. those using no-treatment controls. Differences between subgroups were assessed using the *χ*^2^ test for subgroup interaction (*P* < .10 considered significant). To explore the influence of incontinence subtype, subgroup analyses were also conducted where feasible, categorizing studies by predominant population: SUI only vs. mixed UI (MUI) or urgency UI (UUI).

### Assessment of the Certainty of Evidence

The certainty of the body of evidence for each outcome was assessed using the Grading of Recommendations Assessment, Development and Evaluation (GRADE) approach. Two reviewers independently rated the certainty, considering risk of bias, inconsistency, indirectness, imprecision, and publication bias. Disagreements were resolved by consensus. A summary of findings table was generated for the primary outcome (PFM pressure) and all secondary outcomes (PFM strength, involuntary urine loss, number of leakage episodes, and quality of life), presenting the pooled effect estimates, number of participants and studies, and the overall certainty rating (high, moderate, low, or very low).

### Use of Generative Artificial Intelligence

This study used DeepSeek V3 (DeepSeek AI) as a support tool for exploratory analysis and textual review of the results section. The authors reviewed and validated all generated outputs, assuming full responsibility for the final content.

## Results

This review included 8 primary studies that satisfied the eligibility criteria, evaluating the effectiveness of intravaginal and TES in managing UI. Table 1 presents a summary of the key findings from the included clinical trials.

### Study Selection

A total of 3296 records were identified. After removing 654 duplicates, 2642 records underwent title and abstract screening. Of these, 2622 were excluded as not meeting the eligibility criteria, and 20 full-text reports were assessed. Following full-text review, 12 reports were excluded (reasons detailed in Figure 1), leaving 7 studies included in this systematic review. It is important to note that the reports by Amaro et al^10^ and Amaro et al^14^ originate from the same patient population and were therefore treated as a single study, in accordance with PRISMA guidelines that distinguish between reports and studies.

### Individual Study Results

#### Characteristics of the Studies

Most of the studies were conducted in Brazil, including those by Amaro et al,^10^ Amaro et al,^14^ Barroso et al,^8^ Castro et al,^15^ and Correia et al.^16^. Two of the studies were conducted in the United States and one in Norway.^13^^,^^17^^,^^18^

The placebo group as a comparator was used in the studies by Amaro et al,^10^ Amaro et al,^14^ Barroso et al,^8^ Brubaker et al,^17^ and Sand et al.^13^ The studies by Bø et al,^18^ Castro et al,^15^ and Correia et al^16^ used a control group with no treatment. All the studies carried out assessments at baseline and immediately after the end of treatment, which lasted from 7 weeks to 6 months.^10^^,^^14^^,^^15^^,^^18^

The study population consisted of women presenting with various types of UI, totaling 505 unique participants across 7 studies. The reports by Amaro et al^10^ and Amaro et al^14^ describe the same cohort of 40 women, with different outcome measures; therefore, they were counted as a single study to avoid double-counting. The study by Brubaker et al^17^ included women with UI caused by detrusor instability (UUI), SUI, and MUI, as did Barroso et al.^8^ The studies by Amaro et al^10^^,^^14^ focused exclusively on MUI. In turn, the studies by Sand et al,^13^ Bø et al,^18^ Castro et al,^15^ and Correia et al^16^ investigated women with SUI.

#### Evaluation Tools

In 4 of the included studies, PFM pressure was measured using a perineometer.^10^^,^^13^^,^^16^^,^^18^ Pelvic floor muscle strength was evaluated using the Oxford grading scale and the vaginal cone weight test.^10^^,^^15^^,^^16^

The number of voids was assessed by a voiding diary by Sand et al,^13^ Brubaker et al,^17^ Bø et al,^18^ Barroso et al,^8^ Amaro et al,^10^ and Castro et al.^15^ Voiding frequency was assessed using the voiding diary in 4 studies.^8^^,^^14^^,^^15^^,^^17^

Quality of life was assessed by self-report in 4 studies, using different instruments. Sand et al^13^ used the Short Form Health Survey 36 Questionnaire, while Brubaker et al^17^ used an unvalidated instrument. Castro et al^15^ applied the Incontinence Quality of Life (I-QOL) Questionnaire, and Correia et al^16^ used the King’s Health Questionnaire (KHQ).

The amount of urine lost was assessed in the studies by Sand et al,^13^ Bø et al,^18^ Correia et al,^16^ Amaro et al,^14^ and Castro et al,^15^ all using the PAD test.

#### Frequency and Time of Intervention

The intervention protocols adopted in the studies analyzed showed significant variations in terms of the frequency of sessions, the length of each session, and the total duration of the treatment.

In the studies by Sand et al^13^ and Brubaker et al^17^ followed similar protocols with regard to the frequency and duration of the sessions, with treatments carried out twice a day, each session lasting 20 minutes. However, there was a difference in the total treatment time. While Sand et al^13^ conducted the protocol for 12 weeks, the study by Brubaker et al^17^ lasted 8 weeks. The protocol by Barroso et al^8^ also adopted a frequency of twice a day, with 20 minutes per session, over 12 weeks.

Conversely, the study by Bø et al^18^ adopted a more intensive approach, with sessions held daily (7 times a week), each lasting 30 minutes, totaling 6 months of treatment. This strategy reflects a more rigorous and prolonged protocol, which seeks to ensure greater stimulation and cumulative effect.

In contrast, the studies by Amaro et al^10^^,^^14^ opted for a shorter periodicity, carrying out the sessions 3 times a week, each lasting 20 minutes, for a total period of 7 weeks. This strategy aims to provide regular treatment intervals without overloading the patients.

The study by Castro et al^15^ also used a frequency of 3 times a week, with 20 minutes per session, but with a significantly longer total duration of 6 months. This choice suggests a long-term approach, similar to the protocol of Bø et al.^18^ Finally, in the study by Correia et al,^16^ a total of 12 sessions were held, twice a week, each lasting 20 minutes.

#### Electrostimulation Parameters

The electrostimulation protocols used in the studies analyzed show significant variations in parameters, including frequency, pulse duration, current intensity, and duty cycle Table 2.

In the study by Sand et al,^13^ a frequency of 50 Hz was applied, with a pulse duration of 0.3 ms and current ranging from 0 to 100 mA, adjusted to 60 to 80 mA or the maximum tolerable level. The duty cycle was progressive, alternating stimulation patterns of 5 seconds on/10 seconds off and 5 seconds on/5 seconds off were used.

In contrast, the study by Brubaker et al^17^ used a lower frequency of 20 Hz, with a duty cycle of 2 seconds on/4 seconds off and a pulse width of 0.1 ms. The current varied from 0 to 100 mA, also adjusted to the maximum tolerable level. The difference in frequency and duty cycle compared to Sand’s study suggests a less intense approach, but with a focus on comfort and individual adaptation.

The protocol of Bø et al^18^ used a different approach, with a frequency of 50 Hz and a pulse duration of 0.2 ms, applying an intermittent biphasic current ranging from 0 to 120 mA. The work cycle was adjusted according to the progress of muscle strength, ranging from 0.5 to 10 seconds on and 0 to 30 seconds off.

The study by Barroso et al^8^ adopted a portable device with a variable frequency ranging from 20 to 50 Hz and a pulse width of 300 µs were applied, with an adjustable current of 0 to 100 mA. The duty cycle was 5 seconds on/5 seconds off, preceded by a rise time of 1 second.

The studies by Amaro et al^10^^,^^14^ used a significantly lower frequency of 4 Hz, with a duty cycle of 2 seconds on/4 seconds off and a pulse width of 0.1 µs. The current varied from 0 to 100 mA, using a bipolar square wave. The choice of these parameters suggests a focus on stimulating slower, deeper contractions of the PFMs.

O protocol by Castro et al^15^ used a frequency of 50 Hz, with a pulse width of 0.5 ms and an adjustable current of 0 to 100 mA. The duty cycle was 5 seconds on/10 seconds off, similar to Sand’s study, but with a longer pulse width, which may influence the patient’s perception of intensity and comfort during stimulation.

Finally, the study by Correia et al^16^ adopted a frequency of 50 Hz, with a pulse duration of 700 µs and a cycle of 4 seconds on/8 seconds off, adjusting the intensity to the maximum tolerable level.

### Analysis of the Risk of Bias

The risk of bias assessment indicated that just over 25% of the studies had a high risk of bias in the randomization process, which impacted the overall risk of bias. Deviations from the intended intervention and missing outcome data were rated as low risk in 80% of studies and some concern in 20%. Outcome measurement showed a low risk of bias in 100% of the studies. The selection of reported results was rated as low risk in approximately 40% of studies and some concern in 60%. Overall, 28.6% of studies were classified as low risk (2/7), 42.8% as some concern (3/7), and 28.6% as high risk (2/7). The 2 Amaro et al^10^^,^^14^ reports were treated as a single study, with the higher risk classification (high risk) retained for the pooled assessment (Figure 2).

Figure 3 illustrates the risk of bias assessment for the studies included in this systematic review, highlighting methodological quality across the 6 RoB 2 domains. Regarding the randomization process, most studies were rated as low risk of bias, with the exception of Amaro et al^10^ and Amaro et al^14^, who were classified as high risk, and Castro et al^15^ with some concerns. As for the deviation from the intended intervention, only Castro et al^15^ had some concerns; the rest were considered low risk, ensuring that the interventions were applied as planned. For missing outcome data, Bø et al^18^ and the other studies were classified as having a low risk of bias, indicating that the losses did not compromise the results. In the area of measuring the outcome, all the studies were low risk. The selection of results reported in Amaro et al,^10^ Amaro et al,^14^ Barroso et al,^8^ and Castro et al^15^ raised some concerns, and in Bø et al,^18^ Correia et al,^16^ and Sand et al,^13^ low risk of bias was noted. Finally, in the general assessment of bias, Amaro et al^10^ and Amaro et al^14^ were classified as having a high risk of bias, with some concerns in Barroso et al,^8^ Bø et al,^18^ and Castro et al,^15^ with a low risk of bias in Correia et al^16^ and Sand et al.^13^

### Results—Meta-analysis

### Outcome Pelvic Floor Muscle Contraction Pressure

Four trials, (n = 168) contributed to the meta-analysis of PFM pressure.^10^^,^^13^^,^^16^^,^^18^ After converting mm Hg to cm H_2_O (see Methods), the pooled mean difference (MD) was 3.92 cm H_2_O (95% CI, 1.50-6.34; *P* = .001; *I*^2^ = 55%). Subgroup analysis by control type did not reveal a significant interaction (*P* = .32). In the sham/placebo subgroup, the MD was 2.81 cm H_2_O (95% CI, –0.16-5.78; *P* = .06; *I*^2^ = 0%).^13^^,^^14^ In the no-treatment subgroup, the MD was 5.50 cm H_2_O (95% CI, 2.01-9.00; *P* = .002; *I*^2^ = 0%).^16^^,^^18^ Although the effect appeared larger in studies with no-treatment controls, the difference between subgroups was not statistically significant. The overall intervention effect remained significant regardless of control type.

In a subgroup analysis by incontinence type, the 3 studies that exclusively enrolled women with SUI showed a MD of 5.50 cm H_2_O (95% CI, 2.01-9.00; *P* = .002; *I*^2^ = 0%), while the single study in MUI gave a MD of 1.50 cm H_2_O (95% CI, –3.24-6.24; *P* = .53).^10^^,^^13^^,^^16^^,^^18^ The interaction test was not statistically significant (*P* = .18), but the comparison is limited by the small number of studies and the imbalance between subgroups. Therefore, the overall estimate should be interpreted with caution, as it pools distinct clinical populations.

### Outcome Pelvic Floor Muscle Strength

The meta-analysis for the PFM strength outcome included data from 3 clinical trials, including 61 participants in the experimental group and 59 in the control group.^10^^,^^15^^,^^16^ The greater the MD, the greater the improvement in PFM strength. The pooled effect estimate for this outcome across the studies was 0.89 (95% CI [0.37, 1.41]; *P* = .0008; *I*^2^ = 52%) (Figure 4). Heterogeneity was moderate (*I*^2^ = 52%), indicating some variation between studies, but without compromising the robustness of the findings. Thus, the intervention demonstrated a significant improvement in PFM strength compared to the control group.

### Objective Involuntary Urine Loss Outcome

The meta-analysis results for the outcome involuntary urine loss measured by the Pad Test included 5 clinical trials, with 115 participants in the experimental group and 105 in the control group.^13^^-^^16^^,^^18^ The smaller the MD, the lower the urinary loss. The pooled effect estimate across the studies for the outcome involuntary urine loss was 3.68 g (95% CI [-13.01, 20.38]; *P* = .67; *I*^2^ = 97%) (Figure 5). Heterogeneity was high (*I*^2^ = 97%), indicating great variation between studies, which may compromise the reliability of the combined estimate. The overall effect was not statistically significant (Z = 0.43; *P* = .67), indicating that the intervention did not have a significant impact on reducing urinary loss compared to the control group.

### Outcome Number of Episodes of Urinary Loss

The meta-analysis for the outcome of frequency of urinary leakage episodes included data from 4 clinical trials, comprising 104 participants in the experimental group and 82 in the control group.^8^^,^^13^^,^^15^^,^^18^ Because the included studies reported total counts over diary periods of varying durations (24 hours, 3 days, or 7 days), all values were converted to a common unit of episodes per day prior to pooling (see Methods). The pooled MD was −0.41 episodes per day (95% CI, −0.78 to −0.03; *P* = .03; *I*^2^ = 73%) (Figure 6), indicating a modest reduction in the daily frequency of leakage episodes.

To explore potential sources of heterogeneity, subgroup analyses were conducted by diary duration and incontinence type. When grouped by diary duration, the effect estimates were: 24-hour diary: MD, −0.25 episodes/day (95% CI, −0.55 to 0.05; *P* = .10; *I*^2^ = 0%); 3-day diary: MD, −0.33 episodes/day (95% CI, −0.82 to 0.15; *P* = .18; *I*^2^ not applicable); and 7-day diary: MD, −0.77 episodes/day (95% CI, −1.58 to 0.05; *P* = .07; *I*^2^ = 79%). The difference between subgroups was not statistically significant (*P* = .53).

Subgroup analysis by incontinence type showed that studies confined to SUI^13,15,18^ yielded a MD of −0.46 episodes/day (95% CI, −0.96 to 0.04; *P* = .07; *I*^2^ = 68%), while the single study including mixed incontinence types ^^8^^ gave a MD of −0.17 episodes/day (95% CI, −0.29 to −0.05; *P* = .006). The difference between subgroups was not significant (*P* = .31). These exploratory analyses should be interpreted with caution given the small number of studies.

Overall heterogeneity remained substantial (*I*^2^ = 73%), indicating considerable variability across studies that could not be explained by diary duration or incontinence subtype. The pooled estimate suggests a possible benefit of electrical stimulation on daily leakage frequency, but the evidence was rated as very low certainty (GRADE), downgraded for high heterogeneity, risk of bias, and imprecision. The diary duration subgroups represent different measurement windows rather than post-intervention follow-up times; therefore, no conclusions about temporal sustainability can be drawn from these analyses.

A subgroup analysis was conducted based on follow-up duration (24 hours, 3 days, and 7 days). In the 24-hour subgroup, the effect estimate was −2.80 (95% CI, [−3.75, −1.85]; *P* < .00001), indicating a significant effect. For the 3-day subgroup, the MD was −1.00 (95% CI [−2.45, 0.45]; *P* = .18), showing no statistical significance. In the 7-day subgroup, the effect estimate was −5.36 (95% CI [−11.09, 0.37]; *P* = .07), suggesting a trend toward reduction, although it did not reach statistical significance.

Additional subgroup analysis by type of incontinence showed that studies confined to SUI generated a MD of –2.50 (95% CI, –4.80 to –0.20; *P* = .03; *I*^2^ = 68%), whereas the single study including both SUI and UUI/MUI gave a MD of –4.10 (95% CI, –6.90 to –1.30; *P* = .004).^8^^,^^13^^,^^15^^,^^18^ The difference between subgroups was not significant (*P* = .54). These findings should be viewed as exploratory given the sparsity of data.

Overall heterogeneity was moderate to high (*I*^2^ = 73%), with the greatest variation observed in the 7-day subgroup (*I*^2^ = 79%), suggesting potential methodological or population differences among the studies. The difference between subgroups was not statistically significant (*I*^2^ = 61.8%; *P* = .07), indicating that the effect may be relatively consistent over time. Although the pooled estimate for the number of leakage episodes showed a statistically significant reduction (–2.85 episodes; *P* = .002), the evidence was of very low certainty, downgraded for high heterogeneity (*I*^2^ = 73%) and indirectness.

### Quality of Life Outcome

The meta-analysis for quality of life included 2 trials (41 experimental, 39 control).^15^^,^^16^ Because Castro et al used the I-QOL (higher scores indicate better quality of life) and Correia et al used the KHQ (higher scores indicate worse quality of life), the KHQ scores were inverted (100 – original score) so that higher values uniformly represent improvement. The SMD Hedges’ g was calculated using a random-effects model. The pooled SMD was 0.28 (95% CI, –0.42 to 0.98; *P* = .43; *I*^2^ = 85%) (Figure 7), indicating no statistically significant effect. Sensitivity analysis leaving one study out did not appreciably alter the estimate. The overall certainty of evidence for quality of life was rated as very low (GRADE), owing to imprecision, inconsistency, and high risk of bias

The included trials enrolled women with different incontinence subtypes: 2 studies included only MUI, 4 included only SUI, and 2 included mixed populations.^8^^,^^10^^,^^13^^-^^18^ For the outcomes of PFM strength, involuntary urine loss, and quality of life, the number of studies per subtype was insufficient to perform meaningful subgroup analyses. Consequently, the pooled estimates combine populations with potentially distinct pathophysiologies, and this clinical heterogeneity likely contributes to the high statistical heterogeneity observed.

### Certainty of the Evidence (Grading of Recommendations Assessment, Development, and Evaluation)

Table 3 summarizes the GRADE assessment for all outcomes. The certainty of evidence was rated as low for PFM pressure and PFM strength, and very low for involuntary urine loss, frequency of leakage episodes, and quality of life. Downgrading was primarily due to high risk of bias (lack of blinding, inadequate randomisation in some studies), serious inconsistency (*I*^2^ ranging from 55% to 97%), indirectness (mixed incontinence subtypes and varied control interventions), and imprecision (small total sample sizes and wide CIs).

## Discussion

This systematic review pooled data from 8 randomized trials to evaluate the effectiveness of intravaginal and TES for UI. The meta-analysis revealed a statistically significant increase in PFM pressure and strength, suggesting a short-term physiological effect on PFM function. However, the certainty of this evidence is low (GRADE), meaning that further research is likely to change the estimates. For the patient-important outcomes of involuntary urine loss and quality of life, no statistically significant effects were observed, and the evidence was rated as very low certainty. The extremely high heterogeneity in these latter outcomes (*I*^2^ > 85%) precludes any firm quantitative conclusion and indicates that pooled estimates should be interpreted with extreme caution.

The marked discrepancy between the apparent physiological benefits (pressure and strength) and the absence of significant effects on incontinence severity and quality of life may stem from several sources. Muscle-strengthening outcomes directly capture the biomechanical target of electrical stimulation, whereas urinary loss and well-being are influenced by multiple biomechanical, neuromuscular, and psychosocial factors.^19^^,^^20^ Moreover, the wide variation in stimulation parameters (Table 2), the clinical heterogeneity of participants (SUI, UUI, MUI), and the use of different control conditions (sham vs. no treatment) likely contributed to the very high between-study inconsistency. Hence, the observed benefits in muscle function cannot be extrapolated to clinically meaningful outcomes without robust evidence, which is currently lacking. The standardized mean difference (SMD, Hedges’ g) was calculated using a randomeffects model. The pooled SMD was 0.28 (95% CI –0.42 to 0.98; *P* = .43; I^2^ = 85%) (Figure 8), indicating no statistically significant effect.

The sensitivity of the assessment methods may also have influenced the findings. While strength and muscle pressure were measured objectively, urinary loss and quality of life depend on external factors, such as adherence to treatment and the subjective perception of the participants. In addition, the outcomes may respond to the intervention at different follow-up times. Improvements in muscle function can occur quickly, while reductions in urinary loss and perceived improvements in quality of life can take longer to manifest. The heterogeneity in diary durations across studies (ranging from 24 hours to 7 days) precludes any conclusion about the sustainability of the effect over time. These durations represent different measurement windows, not sequential follow-up assessments. Future studies should adopt standardized diary periods to facilitate meta-analytic pooling and enable more robust inferences about the durability of treatment effects.

Another important consideration is that the perception of quality of life and UI is influenced not only by the physiological effectiveness of the intervention, but also by individual expectations, psychological factors, and social context, as well as psychosocial factors such as adaptation to the problem, emotional impact, and individual expectations.^20^^,^^21^ This variability in personal perception and contextual factors may have contributed to the high heterogeneity observed and the lack of statistical significance in the quality of life outcome. In summary, the results suggest that the intervention analyzed is effective for strengthening the PFMs. Although it clearly improves PFM pressure and strength, its effect on actual UI episodes and patients’ quality of life remains uncertain. The variability of the findings reinforces the need for more standardized clinical trials, with greater methodological rigor and longer follow-up periods, in order to elucidate the effect of the intervention on UI and quality of life in the long term. It was also analyzed that, with regard to the episode of urine loss, a significant positive effect was shown, although with moderate-high heterogeneity, implying that the results may vary substantially between the different studies carried out.

Urinary incontinence is known to cause social isolation and impaired quality of life.^18^ In women, this condition can profoundly disrupt daily activities and personal well-being. Consequently, employing quality of life instruments tailored to this specific dysfunction is critical for a precise assessment of how urinary symptoms affect patients.^22^

Although 2 individual trials have suggested subjective benefits, the present pooled analysis found no statistically significant improvement in quality of life (SMD = 0.28; *P* = .43). The extremely high heterogeneity (*I*^2^ = 85%) and the opposing directions of effect highlight that the current evidence is inconclusive. Methodological differences, including the use of distinct instruments (I-QOL vs. KHQ) and variable follow-up periods, likely contributed to the observed inconsistency. Therefore, claims that electrical stimulation reliably improves quality of life cannot be substantiated by the current meta-analytic evidence. Some preliminary studies have reported subjective improvements in well-being after low-frequency electrostimulation; however, these findings are based on small, uncontrolled series, and are not corroborated by the pooled analysis.^23^ The evidence for urinary loss and quality of life is of very low certainty, primarily due to high heterogeneity, risk of bias, and imprecision. Therefore, no reliable quantitative conclusion can be drawn.

Some uncontrolled studies have reported subjective improvements in symptom perception and well-being; however, these findings are not corroborated by the pooled results and should be interpreted as preliminary.^24^

With regard to involuntary urine loss, the absence of a statistically significant effect can be explained by a series of methodological and clinical factors observed in the studies analyzed. A primary factor contributing to this variability in outcomes is the heterogeneity of the methodologies employed to quantify urinary loss. While some studies used absorbent pad tests to measure urine loss before and after standardized physical activity, others, such as Chen et al and Sand et al^13^^,^^14^^,^^16^^,^^18^^,^^19^ were based on 24-hour voiding diaries and weekly records of leakage episodes. This heterogeneity in the protocols may have made it difficult to identify a uniform effect of the intervention.

Additionally, the study population exhibited variability in participants’ baseline bladder volume. While Correia et al^16^ standardized the intake of 500 ml of water before the test, Bo et al^18^ used instillation of 200 ml of saline solution into the bladder. These methodological variances could have modulated PFM responsiveness and intra-abdominal pressure dynamics during the intervention, thereby affecting urinary leakage outcomes and the overall study results.

Another relevant factor was the discrepancy in the intensity and duration of the provocative exercises. While some studies adopted longer periods, associating physical exercise with the 60-minute absorbent test.^14^^,^^16^^,^^19^ Bo et al^18^ used short, intense efforts, such as the protocol that involved running in place and jumping jacks for just 1 minute. These variations may have had an impact on the sensitivity of the tests, making it more difficult to detect small differences in urinary loss.

In addition to the methodological differences, the clinical heterogeneity of the participants may also have contributed to the lack of statistical significance. The studies included women with different degrees of UI, which may have generated a wide variation in the results. Some participants may have had minimal leakage, while others had substantial leakage, diluting a possible effect of the intervention. Given these factors, the lack of statistical significance in the involuntary urine loss outcomes may be more related to the methodological limitations of the studies than to the actual absence of a clinical effect. Collectively, these findings underscore the necessity for further investigation to elucidate the multifaceted factors influencing quality of life in this patient population. The type of control intervention varied across trials: 4 studies employed a sham/placebo device, while 3 used a no-treatment control. This difference could introduce varying degrees of performance and expectation bias. Subgroup analyses for each outcome did not detect statistically significant differences between control types; however, the low power of these subgroup comparisons and the potential for residual confounding should be acknowledged. Consequently, the certainty of evidence was downgraded for indirectness in the GRADE assessment, as the comparator does not represent a uniformly inert or blinded condition. Given the low to very low certainty of evidence for the outcomes of urinary loss and quality of life, any recommendation for clinical practice should be deferred until larger, methodologically rigorous trials with standardized, validated instruments and longer follow-up are available.

Importantly, the study population was clinically heterogeneous, comprising women with SUI, UUI, and MUI. The mechanisms by which electrical stimulation improves continence may differ between these subtypes. For instance, by enhancing urethral closure in SUI versus inhibiting detrusor overactivity in UUI. The subgroup analyses for PFM pressure and leakage episodes did not detect significant effect modification by incontinence type, but the analyses lacked statistical power and were limited to a few studies. Future trials should either focus on a single incontinence subtype or be designed with sufficient sample size to allow stratified analyses, thereby clarifying whether the intervention’s efficacy differs by clinical presentation. The low to very low certainty of the evidence underscores the necessity for future well-designed trials with adequate sample sizes and standardized protocols, as stated in the conclusion.

Taken together, the currently available evidence indicates that intravaginal/TES can improve surrogate outcomes of PFM function (pressure and strength) in the short term. However, for the patient-centered outcomes of incontinence severity and quality of life, the evidence is inconclusive and of very low certainty. The substantial methodological and clinical heterogeneity, small sample sizes, and short follow-up of existing trials severely limit the generalizability of the findings. Consequently, well-designed, adequately powered randomized controlled trials with standardized protocols, validated outcome instruments, and longer follow-up are urgently required before this intervention can be recommended in routine clinical practice.

## Conclusion

The intervention demonstrated efficacy in enhancing PFM strength and pressure. A modest reduction in the daily frequency of urinary leakage episodes was observed when data were standardized to a common unit, though this finding is based on very low certainty evidence and should be interpreted with caution. However, the certainty of this evidence is low, meaning that further research is likely to change these estimates. Its effects on the objective reduction of UI and on long-term quality of life remain inconclusive, and the clinical diversity among included populations precludes firm recommendations for specific incontinence subtypes. The substantial heterogeneity observed across several outcome measures further underscores the necessity for future randomized clinical trials with enhanced methodological rigor. The GRADE assessment indicates low to very low certainty for all outcomes, reinforcing that current evidence is insufficient to support definitive clinical recommendations.

## Supplementary Materials

Supplementary Material

## Figures and Tables

**Figure 1.**
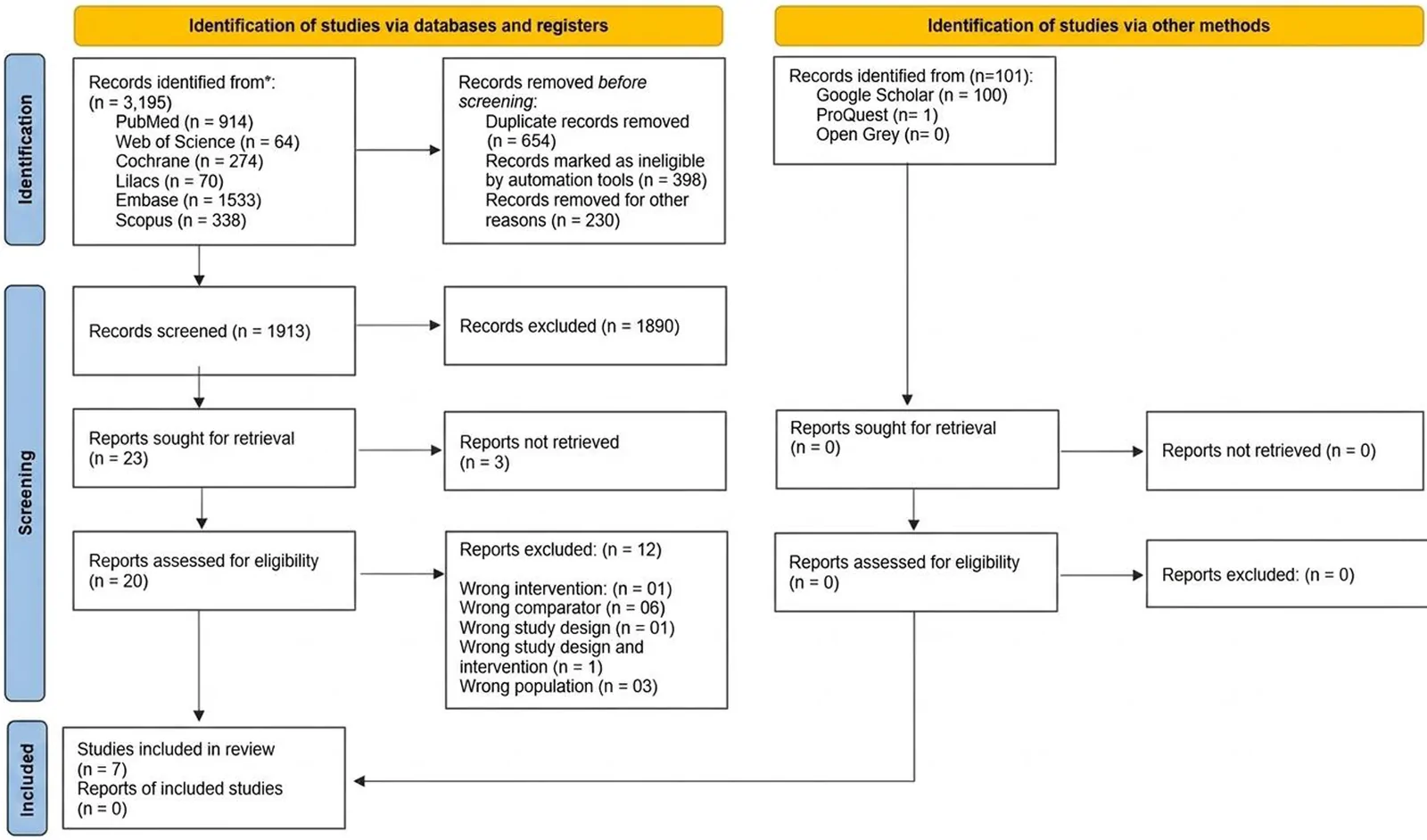
PRISMA 2020 flow diagram for systematic reviews. One report (Amaro et al, 2006) was excluded because it reports on the same patient population as Amaro et al (2005). PRISMA, Preferred Reporting Items for Systematic Reviews and Meta-Analyses.

**Figure 2.**
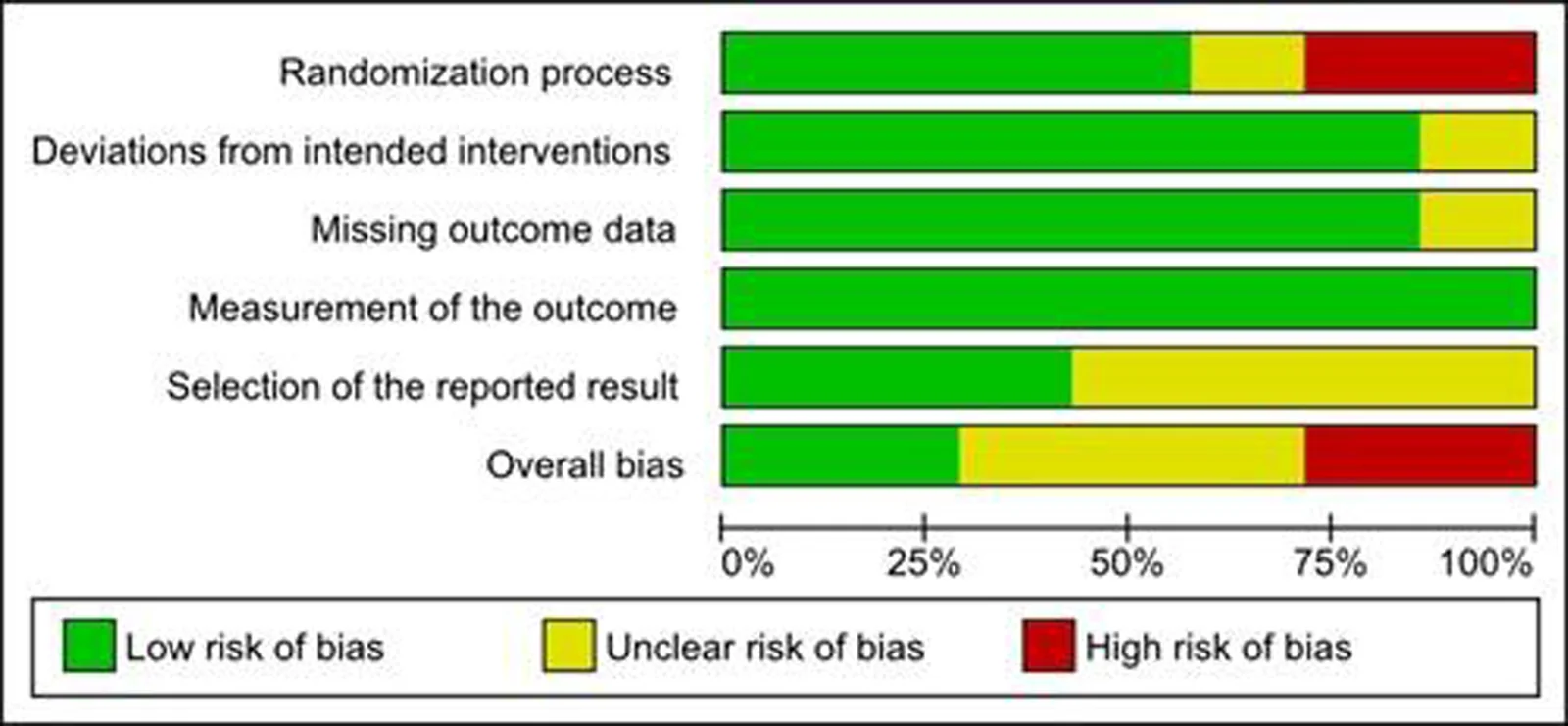
Review authors’ assessments of each risk of bias domain for all included studies.

**Figure 3.**
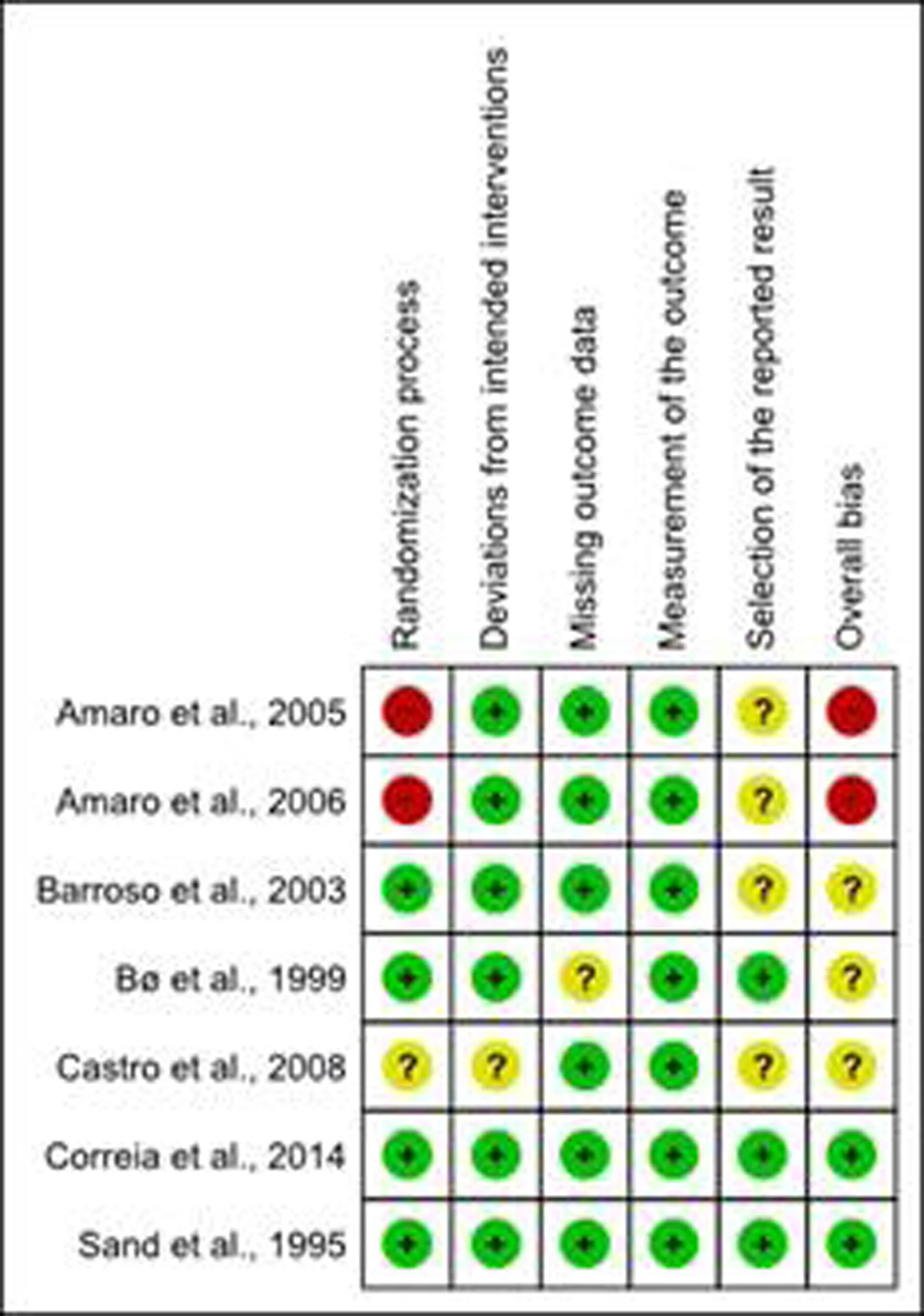
Review authors’ assessments of each risk of bias domain, presented as percentages across all included studies.

**Figure 4.**
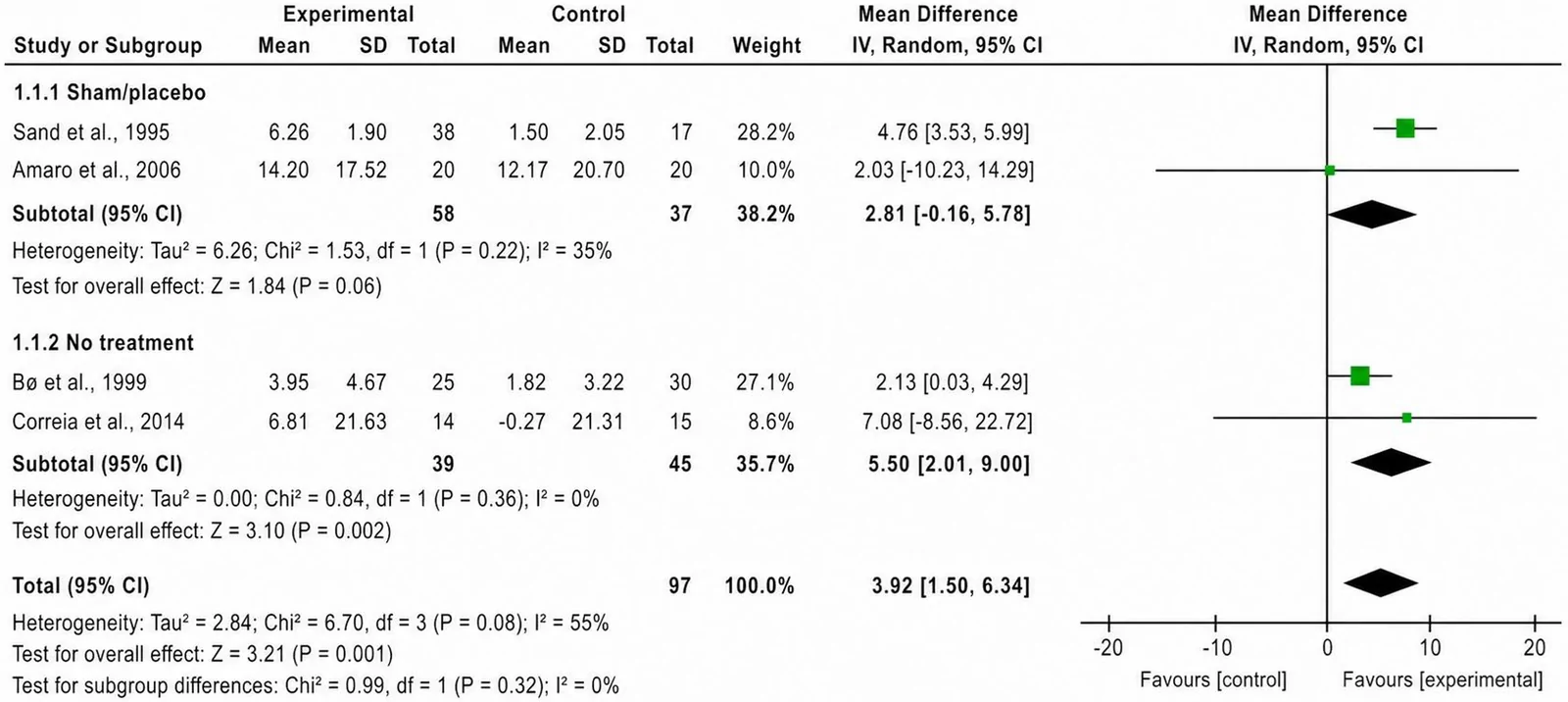
Forest plot of comparison experimental vs control—outcome PFM strength. PFM, pelvic floor muscle.

**Figure 5.**
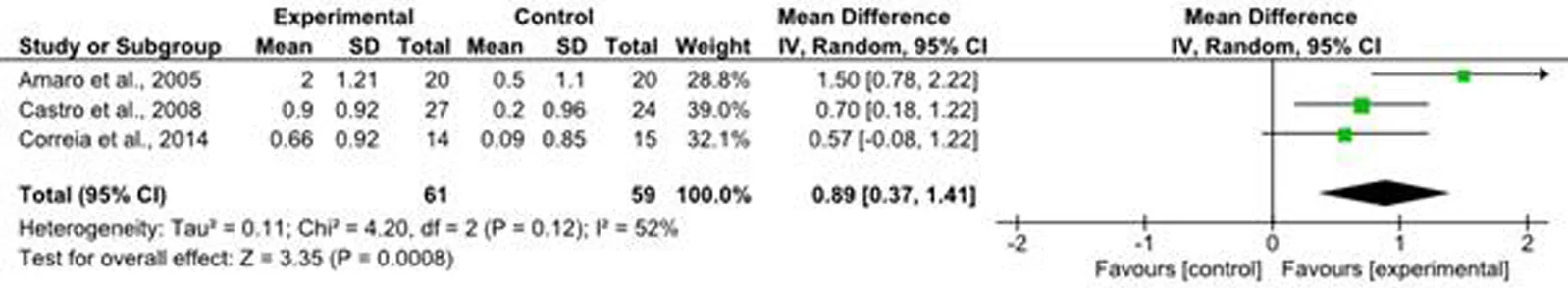
Forest plot of comparison experimental vs control—outcome urinary leakage.

**Figure 6.**
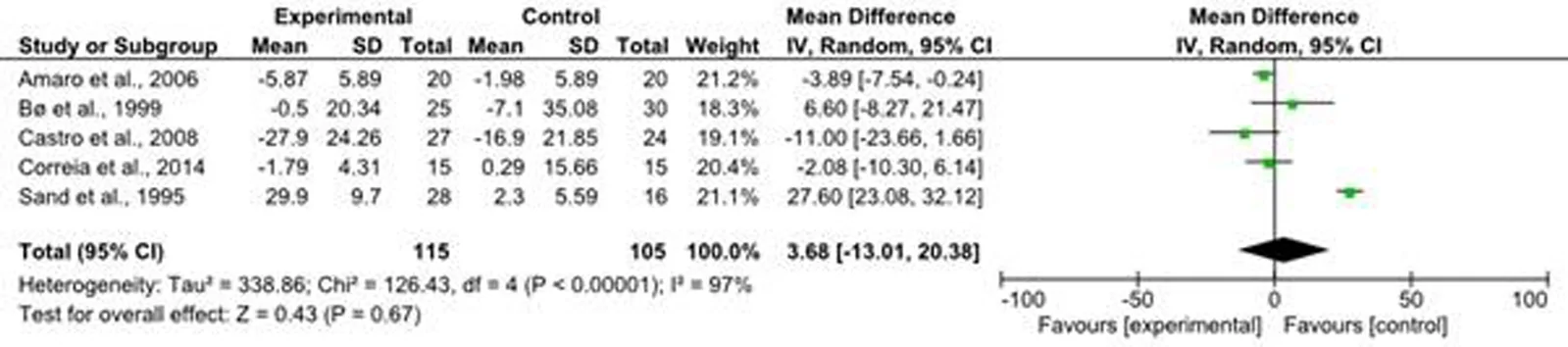
Forest plot of comparison: experimental vs. control—outcome: frequency of urinary leakage episodes (episodes/day). All values were converted to a common daily unit. Diary durations: 24 hours (Sand et al, 1995; Barroso et al, 2004), 3 days (Bø et al, 1999), 7 days (Castro et al, 2008). Subgroup analyses by diary duration are exploratory and do not represent temporal follow-up.

**Figure 7.**
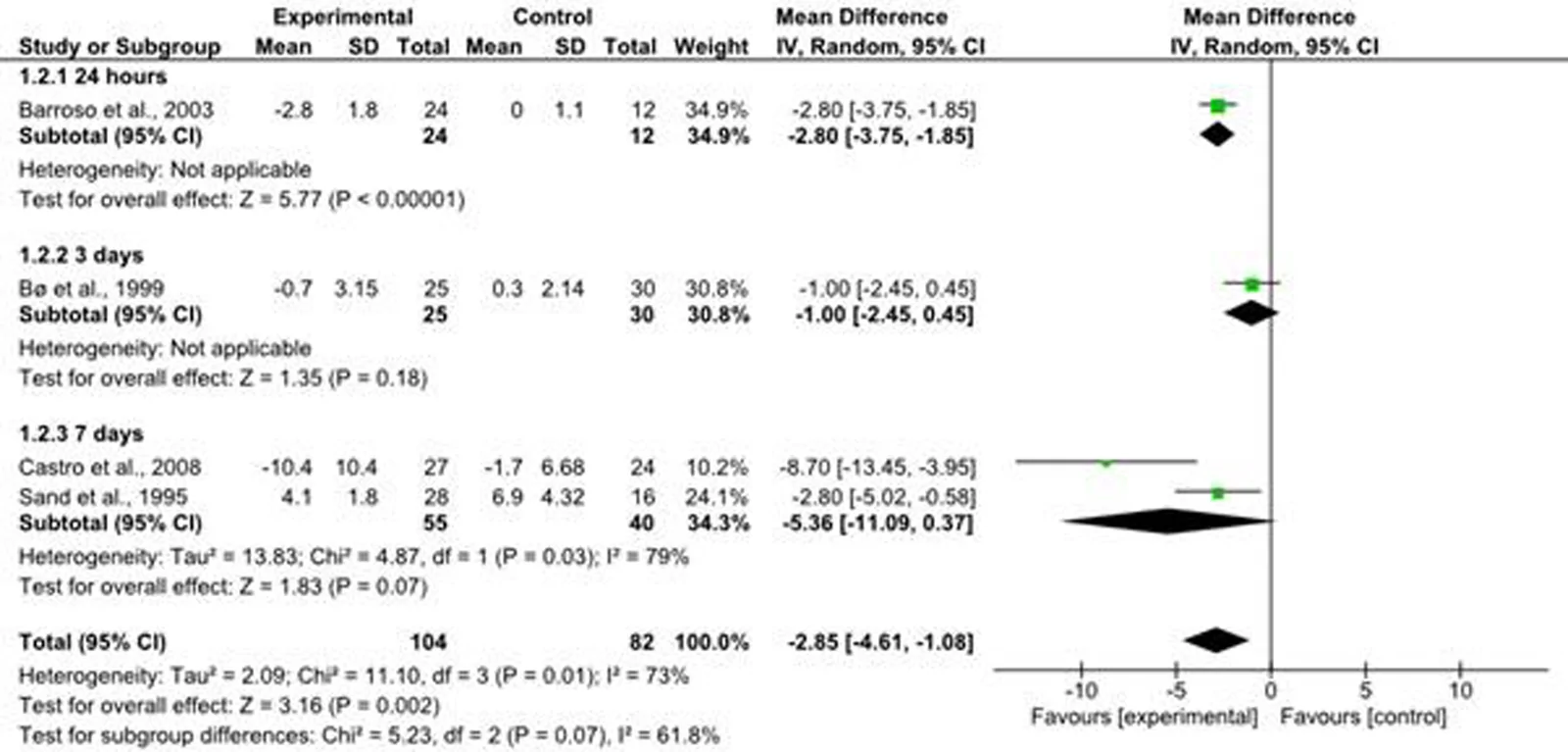
Forest plot of comparison: experimental vs control—outcome: quality of life (SMD, random-effects). I-QOL scores used as reported; KHQ scores inverted (100 – original). I-QOL, Incontinence Quality of Life Questionnaire; KHQ, King’s Health Questionnaire; SMD, standardized mean difference.

**Figure 8.**
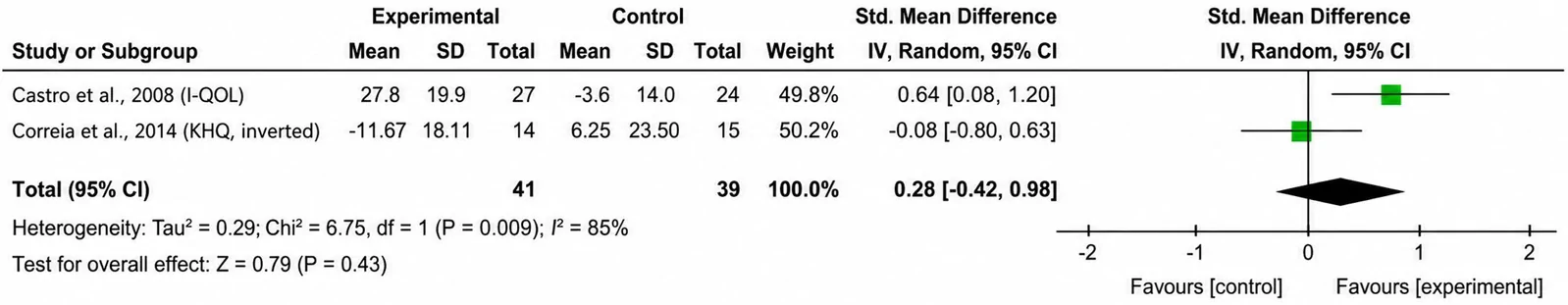
Forest plot of PFM contraction pressure (cm H_2_O): experimental vs. control, subgrouped by control type. Mean differences > 0 favor the intervention. Subgroup differences: *P* = .32. Overall effect: 3.92 cm H_2_O (95% CI, 1.50-6.34; *P* = .001; *I*^2^ = 55%). Studies: (a) SUI: Bø et al, 1999, Correia et al, 2013, Sand et al, 1995; (b) MUI: Amaro et al, 2005. Subgroup interaction test *P* = .18. MUI, mixed urinary incontinence; PFM, pelvic floor muscle; SUI, stress urinary incontinence.

**Table 1.** Summary Findings of the Studies

Eligible Studies	Country	Study Design	Sample Description	Intervention Protocol	Time Point	Outcome/Measuring Instruments	Conclusion
Amaro et al, 2006	Brazil	Randomized, double-blind clinical trial	N = 40 EG: (n = 20) PG: (n = 20) Age: EG 49; PG 47	EG: IES PG: Sham	T0: baseline T1: post-intervention (7 weeks)	– Number of urinations in 24 h: voiding diary – Urine loss: PAD test	Both groups improved with no significant difference.
Barroso et al, 2003	Brazil	Double-blind randomized controlled clinical trial	N = 36 EG: (n = 24) PG: (n = 12) Age: EG 54; PG 56	EG: IES PG: Sham	T0: baseline T1: post-intervention (12 weeks)	– Number of urinations in 24 h: voiding diary – Urine loss: 24-h voiding diary	TES is an effective and safe alternative for reducing urinary frequency, urgency, and incontinence episodes.
Sand et al, 1995	USA	Multicenter, prospective, randomized, double-blind clinical trial	N = 55 EG: (n = 38) PG: (n = 17) Age: 54	EG: IES PG: Sham	T0: baseline T1: post-intervention (15 weeks)	– PFM pressure: perineometer (mm Hg) – Urine loss: 24-h and 7-day voiding diary; PAD test – Quality of life: SF-36	Significant improvements in leakage episodes, PFM strength, and pad test results.
Bø et al, 1999	Norway	Controlled, randomized, single-blind, stratified clinical trial	N = 107 EG1: (n = 25) PFM exercises EG2: (n = 25) IES EG3: (n = 27) Cones CG: (n = 30) no treatment Age: 49.5	EG2: IES CG: no treatment	T0: baseline T1: post-intervention (6 months)	– PFM pressure: perineometer (cm H_2_O) – PFM strength: vaginal balloon – Urine loss: PAD test; 3-day voiding diary	IES improved PFM strength and moderately reduced leakage; inferior to PFM exercises.
Correia et al, 2013	Brazil	Randomized controlled clinical trial	N = 45 EG1: (n = 15) SES EG2: (n = 15) IES CG: (n = 15) no treatment Age: over 50	EG2: IES CG: no treatment	T0: baseline T1: post-intervention (3 months)	– Urine loss: PAD test – PFM pressure: perineometer (cm H_2_O) – PFM strength: Oxford scale – Quality of life: KHQ	SES and IVES improved QoL, urinary leakage, PFM strength and pressure.
Amaro et al, 2005	Brazil	Randomized, double-blind clinical trial	N = 40 EG: (n = 20) PG: (n = 20) Age: 48	EG: IES PG: Sham	T0: baseline T1: post-intervention (7 weeks)	– PFM pressure: perineometer (cm H_2_O) – PFM strength: vaginal cone – Number of urinations 24 h: voiding diary	Significant improvement in PFM pressure, strength, and number of urinations.
Castro et al, 2008	Brazil	Single-blind, randomized, controlled trial	N = 101 EG1: (n = 26) PFM exercises EG2: (n = 27) TES EG3: (n = 24) cones CG: (n = 24) no treatment Age: n/i	EG2: TES CG: no treatment	T0: baseline T1: post-intervention (6 months)	– Urinary loss: 7-day voiding diary; PAD test (200 ml) – PFM strength: Oxford scale – Quality of life: I-QOL	TES significantly reduced pad test, QoL, and stress incontinence episodes vs control.
Brubaker et al, 1997	USA	Prospective, double-blind, randomized clinical trial	N = 121 EG: (n = 61) PG: (n = 60) Age: 57.7	EG: IES PG: Sham	T0: baseline T1: post-intervention (8 weeks)	– Quality of life: unvalidated instrument – Urinary loss: 24-h voiding diary – Number of urinations 24 h: voiding diary	IES effective for QoL and leakage episodes; no significant difference in voiding frequency.

Age, average years; CG, control group; EG, experimental group; IES, intravaginal electrical stimulation; I-QOL, Incontinence Quality of Life Questionnaire; KHQ, King’s Health Questionnaire; PFM, pelvic floor muscles; PG, placebo group; SES, superficial electrical stimulation; SF-36, Short Form Health Survey 36; TES, transvaginal electrical stimulation.

**Table 2.** Electrotherapy Parameters

Study	Frequency (Hz)	Pulse Width	Current (mA)	Work–Rest Cycle	Intensity
Sand et al (1995)	50	0.3 ms	0-100	5 s on/10 s off and 5 s on/5 s off	60-80 mA or maximum tolerable level
Brubaker et al (1997)	20	0.1 ms	0-100	2 s on/4 s off	Maximum tolerable level
Bø et al (1999)	50	0.2 ms	0-120	0.5-10 s on/0-30 s off	Individually adjusted
Barroso et al (2004)	20-50	300 µs	0-100	5 s on/5 s off, 1 s rise time	Maximum tolerable level
Amaro et al (2005)	4	0.1 µs	0-100	2 s on/4 s off	Maximum tolerable level
Amaro et al (2006)	4	0.1 µs	0-100	2 s on/4 s off	Maximum tolerable level
Castro et al (2008)	50	0.5 ms	0-100	5 s on/10 s off	Maximum tolerable level
Correia et al (2013)	50	700 µs	Maximum tolerable level	4 s on/8 s off	Maximum tolerable level

**Table 3.** Summary of Findings: Intravaginal/Transvaginal Electrical Stimulation Compared to Control for Urinary Incontinence

Outcome	No of Participants (Studies)	Relative Effect (95% CI)	Anticipated Absolute Effect (Intervention vs Control)	Certainty of the Evidence (GRADE)	Comments
PFM pressure (cm H_2_O)	128 (3 RCTs)	–	MD 3.92 cm H_2_O higher	⊕⊕◯◯ Low^1^	Downgraded for risk of bias and inconsistency
PFM strength (various scales)	120 (3 RCTs)	–	SMD 0.89 SD higher (0.37 higher to 1.41 higher)	⊕⊕◯◯ Low^2^	Downgraded for risk of bias and inconsistency
Involuntary urine loss (g/24 h)	220 (5 RCTs)	–	MD 3.68 g higher (13.01 lower to 20.38 higher)	⊕◯◯◯ Very low^3^	High heterogeneity (*I*^2^ = 97%); not significant
Frequency of leakage episodes (episodes/day or week)	186 (4 RCTs)	–	MD 0.41 episodes/day fewer (0.78 fewer to 0.03 fewer)	⊕◯◯◯ Very low^4^	Short-term measurement; substantial heterogeneity (*I*^2^ = 73%); diary durations varied; not interpretable as temporal follow-up^5^
QoL (SMD)	80 (2 RCTs)	–	SMD 0.28 SD higher (0.42 lower to 0.98 higher)	⊕◯◯◯ Very low	Different instruments; not significant^6^

GRADE Working Group grades of evidence:

High certainty: further research is very unlikely to change the confidence in the estimate of effect.

Moderate certainty: further research is likely to have an important impact.

Low certainty: further research is very likely to have an important impact.

Very low certainty: any estimate of effect is very uncertain.

^1^Downgraded one level for risk of bias: some trials had high risk of bias in randomization and lack of blinding.

^2^Downgraded one level for inconsistency: substantial to considerable heterogeneity (*I*^2^ 55%-97%).

^3^Downgraded one level for indirectness: mixed control types (sham vs. no treatment).

^4^Downgraded one level for imprecision: wide CI overlapping no effect and small sample size.

^5^Downgraded one level for imprecision and indirectness: short follow-up and mixed incontinence subtypes.

^6^Downgraded one level for indirectness and imprecision: different QoL scales, opposing directions.

GRADE, Grading of Recommendations Assessment, Development and Evaluation; MD, mean difference; SMD, standardized mean difference; PFM, pelvic floor muscle; QoL, quality of life; RCT, randomized controlled trial.

## Data Availability

The data that support the findings of this study are available on request from the corresponding author.
