# Supplementary material for: Evaluating Intravaginal and Transvaginal Electrical Stimulation Techniques for Urinary Incontinence: A Systematic Review with Meta-analysis
Source: Eurasian J Med. 2026 Oct 16;58(4):e251298. doi: 10.5152/eurasianjmed.2026.251298 (PMC13753934; doi:10.5152/eurasianjmed.2026.251298)
Supplement: Supplementary file 1 [file supplementary_material.pdf]

Appendix 1. Database search strategy

PubMed	#1. "Urinary Incontinence"[Mesh] OR (Incontinence, Urinary)<br>#2. "Electric Stimulation Therapy"[Mesh] OR (Therapy, Electric Stimulation) OR (Stimulation Therapy, Electric) OR (Electrotherapy) OR (Therapeutic Electric Stimulation) OR<br>(Electric Stimulation, Therapeutic) OR (Stimulation, Therapeutic Electric) OR (Electrical Stimulation Therapy) OR (Stimulation Therapy, Electrical) OR (Therapy, Electrical Stimulation) OR (Therapeutic Electrical Stimulation) OR (Electrical Stimulation, Therapeutic) OR (Stimulation, Therapeutic Electrical)<br>#3. ((clinical[Title/Abstract] AND trial[Title/Abstract]) OR clinical trials as topic[MeSH Terms] OR clinical trial[Publication Type] OR random*[Title/Abstract] OR random allocation[MeSH Terms] OR therapeutic use[MeSH Subheading])<br>#4. #1 AND #2 AND #3
LILACS via BVS	#1. MH:"Incontinência Urinária" OR (Incontinência Urinária) OR (Urinary Incontinence) OR (Incontinencia Urinaria) OR MH:C12.050.351.968.934.814$ OR MH:C12.200.777.934.852$ OR MH:C12.950.934.814$ OR MH:C23.888.942.343.800$<br>#2. MH:"Terapia por Estimulação Elétrica" OR (Terapia por Estimulação Elétrica) OR (Electric Stimulation Therapy) OR (Terapia por Estimulación Eléctrica) OR MH:E02.331$ OR MH:E02.779.468$ OR<br>MH:E02.831.535.468$ OR MH:HP3.018.129$<br>#3. #1 AND #2
Cochrane	#1. MeSH descriptor: [Urinary Incontinence] explode all trees (3610)<br>#2. (Incontinence, Urinary) (9612)<br>#3. #1 OR #2 (9612)<br>#4. MeSH descriptor: [Electric Stimulation Therapy] explode all trees (11378)<br>#5. (Electrotherapy, Interferential Current) OR (Interferential Current Electrotherapy) OR (Therapy, Electric Stimulation) OR (Electrotherapy) OR (Electrical Stimulation Therapy) OR (Electrical Stimulation, Therapeutic) OR (Electric Stimulation, Therapeutic) OR (Therapeutic Electrical Stimulation) OR (Therapeutic Electric Stimulation) OR (Stimulation, Therapeutic Electrical); (Therapy, Electrical Stimulation) OR (Stimulation Therapy, Electrical) OR (Stimulation, Therapeutic Electric) OR (Stimulation Therapy, Electric) (16)<br>#6. #4 OR #5<br>#7. #3 AND #6
Web of Science via Clarivate	("Urinary Incontinence") (Topic) AND ("Therapy, Electric Stimulation" OR "Stimulation Therapy, Electric" OR "Electrotherapy" OR "Therapeutic Electric Stimulation" OR "Electric Stimulation, Therapeutic" OR "Stimulation, Therapeutic Electric" OR "Electrical Stimulation Therapy" OR "Stimulation Therapy, Electrical" OR "Therapy, Electrical Stimulation" OR "Therapeutic Electrical Stimulation" OR "Electrical Stimulation, Therapeutic" OR "Stimulation, Therapeutic Electrical" OR "Interferential Current Electrotherapy" OR "Electrotherapy, Interferential Current") (Topic)
SCOPUS	(Incontinence, Urinary) AND ("Therapy, Electric Stimulation" OR "Stimulation Therapy, Electric" OR "Electrotherapy" OR "Therapeutic Electric Stimulation" OR "Electric Stimulation, Therapeutic" OR "Stimulation, Therapeutic Electric" OR "Electrical Stimulation Therapy" OR "Stimulation Therapy, Electrical" OR "Therapy, Electrical Stimulation" OR "Therapeutic Electrical Stimulation" OR "Electrical Stimulation, Therapeutic" OR "Stimulation, Therapeutic Electrical" OR "Interferential Current Electrotherapy" OR "Electrotherapy, Interferential Current")
EMBASE VIA ELSEVIER	#1. 'urine incontinence'/exp OR 'bladder incontinence' OR 'incontinence, urine' OR 'incontinentia urinae' OR 'involuntary urinary loss' OR 'involuntary urination' OR 'involuntary urine loss' OR 'leakage of urine' OR 'unwanted urine loss' OR 'urinary incontinence' OR 'urinary leakage' OR 'urine bladder incontinence' OR 'urine leakage' OR 'wetting, urine' OR 'urine incontinence'<br>#2. 'electrotherapy'/exp OR 'electric stimulation therapy' OR 'electro therapy' OR 'electrostimulation therapy' OR 'therapeutic electric stimulation' OR 'therapeutic electrical stimulation' OR 'therapeutic electro-stimulation' OR 'therapeutic electrostimulation' OR 'electrotherapy'<br>#3. #1 AND #2<br>#4. #3 AND [embase]/lim NOT ([embase]/lim AND [medline]/lim)
ProQuest	("Urinary Incontinence") (Topic) AND ("Therapy, Electric Stimulation" OR "Stimulation Therapy, Electric" OR "Electrotherapy" OR "Therapeutic Electric Stimulation" OR "Electric Stimulation, Therapeutic" OR "Stimulation, Therapeutic Electric" OR "Electrical Stimulation Therapy" OR "Stimulation Therapy, Electrical" OR "Therapy, Electrical Stimulation" OR "Therapeutic Electrical Stimulation" OR "Electrical Stimulation, Therapeutic" OR "Stimulation, Therapeutic Electrical" OR "Interferential Current Electrotherapy" OR "Electrotherapy, Interferential Current") (Topic)
Google Scholar	(Incontinence, Urinary) AND ("Therapy, Electric Stimulation" OR "Stimulation Therapy, Electric" OR "Electrotherapy" OR "Therapeutic Electric Stimulation" OR "Electric Stimulation, Therapeutic" OR "Stimulation, Therapeutic Electric" OR "Electrical Stimulation Therapy" OR "Stimulation Therapy, Electrical" OR "Therapy, Electrical Stimulation" OR "Therapeutic Electrical Stimulation" OR "Electrical Stimulation, Therapeutic" OR "Stimulation, Therapeutic Electrical" OR "Interferential Current Electrotherapy" OR "Electrotherapy, Interferential Current")
Open Grey	("Urinary Incontinence") AND ("Therapy, Electric Stimulation" OR "Stimulation Therapy, Electric" OR "Electrotherapy" OR "Therapeutic Electric Stimulation" OR "Electric Stimulation, Therapeutic" OR "Stimulation, Therapeutic Electric" OR "Electrical Stimulation Therapy" OR "Stimulation Therapy, Electrical" OR "Therapy, Electrical Stimulation" OR "Therapeutic Electrical Stimulation" OR "Electrical Stimulation, Therapeutic" OR "Stimulation, Therapeutic Electrical" OR "Interferential Current Electrotherapy" OR "Electrotherapy, Interferential Current")
